# Clinical and Economic Outcomes of Thyroid Surgery in Elderly Patients: A Systematic Review

**DOI:** 10.1155/2012/615846

**Published:** 2012-06-21

**Authors:** Michael C. Sullivan, Sanziana A. Roman, Julie A. Sosa

**Affiliations:** Department of Surgery, Yale School of Medicine, New Haven, CT 06520, USA

## Abstract

The U.S. population is undergoing a dramatic shift in demographics, with a rise in the proportion of elderly Americans. Given an increased prevalence of thyroid disease and malignancy with age, understanding the safety of thyroid surgery in this age group is increasingly pertinent. There remains disagreement regarding the clinical outcomes of elderly patients after thyroidectomy and the applicability of single-institution cohorts to the population at large. This paper reviews the epidemiology of thyroid disease in the elderly, current surgical indications and practice patterns, and the clinical and economic outcomes of elderly patients with thyroid disease after surgical intervention.

## 1. Introduction

 The U.S. population is rapidly changing. According to Census Bureau projections, the number of elderly Americans will double to 80 million by 2050; the most expeditious growth is expected to occur between 2010 and 2030, when the number of Americans aged >65 years will increase by 2.8% annually [1]. The “oldest old,” or individuals >85 years, are the most rapidly expanding subgroup of elderly citizens. Numbering 3 million in 1994, they represented 10% of the elderly and 1% of the overall population; by 2050, an estimated 19 million individuals >85 years are projected to comprise 24% of elderly Americans, and 5% of the total population [1].

Approximately 50% of all surgical procedures are performed on patients >65 years [2]; according to the National Hospital Discharge Survey, elderly patients represented 39% of discharges from short-stay hospitals and 45% of days of inpatient care in 2009 [3]. Given increasing life expectancy and a generalized expansion of surgical indications, the elderly will likely account for a significant proportion of the anticipated 14–47% increase in demand for surgical services by 2020 [2, 4]. For surgical oncologists, this increase is estimated to be 42.7% and 25.4% for selected inpatient and outpatient procedures, respectively [5].

 The prevalence of thyroid disease and thyroid malignancy increases with age [6–8]. Common surgical indications in the elderly include hyperthyroidism resistant to medical management, symptoms of compression due to retrosternal goiter extension, suspicion of a malignant nodule requiring histologic diagnosis, or thyroid carcinoma [9, 10]. While age itself is not a contraindication for major surgery [8, 10], controversy remains regarding the safety of surgical interventions for thyroid disease in aging Americans.

## 2. Epidemiology

 Thyroid gland dysfunction is common among older patients, and can lead to significant morbidity when left untreated. For hyperthyroidism, the prevalence of overt disease in the elderly is estimated to be 0.5–4%, with subclinical hyperthyroidism having a prevalence of 3–8% [6]. Inciting causes of thyrotoxicosis most frequently include toxic multinodular goiter (MNG) and Graves disease [11–13], with the prevalence of both toxic adenomas and MNG increasing with age [6]. While many patients demonstrate classic signs of sympathetic overactivity, a proportion of elderly patients will present with “masked” or “apathetic” thyrotoxicosis [14, 15]. Nonspecific symptoms that can be attributed to the aging process or previously diagnosed health issues, such as weight loss, muscle weakness, loss of appetite, and apathy, can delay diagnosis and may compromise outcomes [16]. In a cross-sectional study involving 3,049 patients, those >60 years most frequently reported 0–2 symptoms of hyperthyroidism at diagnosis (54.4%, *P* < .001 across all age groups) and were least likely to report ≥5 symptoms (14.8%, *P* < .001 across all age groups) [17].

The prevalence of thyroid nodules increases with age; almost 50% of patients ≥65 years demonstrate nodules on ultrasound examination [18], with a similar prevalence among autopsies performed for the general population [19, 20]. Further, there is an established association between age and the malignant potential of thyroid nodules [21, 22]. Thyroid carcinoma represents approximately 1% of all U.S. cancers, with an estimated 0.1% of adults aged 50–70 years having clinically apparent disease [6]. The Centers for Disease Control (CDC) estimates there will be 56,460 incident cases and 1,780 deaths from thyroid cancer in 2012 [23]. Well-differentiated thyroid cancer (DTC), comprised of papillary, follicular, and Hürthle cell histologic subtypes, accounts for >90% of all diagnoses; poorly differentiated, medullary, and anaplastic thyroid cancers comprise a much smaller percentage of cases [24, 25]. 

While women are afflicted by thyroid cancer two to three times more frequently than men, this gender disparity appears to decline among elderly patients [26, 27]. At the same time, older age is associated with changes in the incidence and behavior of thyroid cancer subtypes. Papillary thyroid cancer behaves more aggressively among elderly patients, with more frequent extrathyroidal spread and distant metastases [28, 29]. Previous research examining 30,504 patients with papillary thyroid cancer from the Surveillance, Epidemiology, and End Results (SEER) registry found that cervical lymph node metastases did not affect survival among patients aged <45 years (*P* = .535), but they conferred a 46% increased risk of death to those individuals ≥45 years (*P* < .001) [30]. Among all differentiated cancers, age >60 years has been associated with an increase in cause-specific mortality and failure of surgical extirpation [31, 32].

For medullary cancer, older age is more closely associated with sporadic than hereditary tumors; it also has been cited as a poor prognostic factor [33]. Anaplastic thyroid cancer represents 2–5% of all thyroid tumors and has a peak incidence in the 6th-7th decades of life [34]. Generally arising in the setting of a goiter or DTC, this disease is almost uniformly fatal.

## 3. Surgical Indications and Practice Patterns

Because of an elevated risk for perioperative morbidity among elderly patients undergoing surgical procedures, indications for thyroidectomy in this population are often restricted to overt compressive symptoms or a strong suspicion for malignancy [8]. Lang and Lo studied patients undergoing total thyroidectomy for MNG and found that 38.2% of elderly patients (age ≥ 70 years) had compressive symptoms as their principal indication for surgery [35]. They were more likely than younger patients to have as an indication thyrotoxicosis (30.9% versus 11.6%, *P* < .001) or recent gland enlargement (27.3% versus 7.9%, *P* < .001). Passler et al. reported that patients ≥75 years were more likely to undergo thyroid surgery for suspected or verified malignancy (52.7% versus 30.3%, *P* < .001) or compressive symptoms (38.2% versus 3.1%, *P* < .001), but significantly less likely to have surgery for a noncompressive benign goiter (9.1% versus 66.6%, *P* < .001) [36]. In neither study did the type/extent of procedure significantly differ between age groups. These findings are partially supported by Rios et al. who reviewed 591 patients (81 patients >65 years) receiving treatment for MNG [8]. Elderly patients were more likely than their younger counterparts to have compressive symptoms (43% versus 21%, *P* = .001) and less likely to have concern for malignancy (19% versus 29%, *P* = .031), recent goiter growth (1% versus 6%, *P* = .021), or patient request (4% versus 12%, *P* = .001). 

 For patients >45 years with DTC that is ≥1 cm in size, current American Thyroid Association (ATA) guidelines recommend near-total or total thyroidectomy with adjuvant radioiodine ablation (RAI) for patients with metastases or a functional thyroid remnant [37]. Park et al. used SEER to describe the treatment patterns of elderly Americans with DTC for which the ATA guidelines would suggest surgery and RAI. Among 8,899 patients (45–64 years: 6,184; 65–79 years: 2,271; ≥80 years: 444), 78% received near-total or total thyroidectomy, 21% lobectomy, and 1% no surgery; 52% of all patients received adjuvant RAI [25]. Patients ≥65 years demonstrated more aggressive disease with multiple, larger tumors and more advanced-stage disease, nonpapillary histology, and extrathyroidal extension (all *P* < .001). Nevertheless, elderly patients were less likely to undergo near-total or total thyroidectomy, lymphadenectomy, or radiation treatment (all *P* < .001). Elderly patients were observed to undergo less aggressive treatment than that recommended by the ATA for all stages of disease; on multivariate analysis, older age was associated with less aggressive surgery (65–79 years: OR—1.27, ≥80 years: OR—2.32) and failure to receive RAI (65–79 years: OR—1.16, ≥80 years: OR—2.45).

 Similar findings have been reported for MTC. Panigrahi et al. retrospectively examined the treatment experiences of 2,033 patients with MTC using SEER [38]. Among all patients (*n* = 1, 344) without local invasion or distant metastases, only 59% underwent total thyroidectomy and central compartment neck dissection, as recommended by the ATA [39]; when stratified by age, older patients were less likely to receive appropriate treatment (age <40 years: 65%, 40–65 years: 62%, >65 years: 45%, *P* < .001). On multivariate regression analysis, age >65 years was independently associated with a patient receiving care that was out of step with ATA guidelines (OR: 3.1); on Kaplan-Meier survival analysis, this was associated with compromised disease-specific survival (*P* < .05) [38].

## 4. Clinical Outcomes

 There is evidence from single institution analyses to support the safety of thyroid surgery in the elderly. Passler et al. reviewed the experiences of 738 patients (55 aged ≥75 years) undergoing thyroid surgery over a 5-year study period [36]. The elderly cohort was more likely to undergo remedial thyroid surgery (18.2% versus 6.7%, *P* = .006). The rate of early postoperative complications (elderly: 25.5% versus younger: 21.8%), including hypocalcemia and recurrent laryngeal nerve (RLN) injury, did not differ between age groups. There was no 30-day mortality. Notably, 12.7% of elderly patients were admitted to the ICU for postoperative care; among all elderly patients, the mean preoperative and total hospital length of stays (LOS) were exceptionally long compared to conventional standards at 4.3 and 14.2 days, respectively. The authors acknowledge that each patient underwent operative intervention because of an “absolute necessity,” thus representing a carefully selected cohort. Seybt et al. compared the post-thyroidectomy experiences of 86 young patients (aged 21–35 years) and 44 elderly patients (aged ≥65 years) [40]. While patient comorbidities and operative details (including the procedure performed) were not provided, the rates of transient postoperative hypocalcemia, true vocal cord paralysis, and complications were similar between age groups. Due to small sample size, the study might not have been adequately powered to detect meaningful differences in outcomes based on age; for example, the elderly cohort demonstrated an almost fourfold higher rate of readmission, but this was not significant (4.5% versus 1.2%, *P* = .26).

 Bliss et al. presented a single institution series of 1,631 patients who underwent 1,673 thyroid procedures; patients were divided into three age groups: 50–60 years (725 patients), 61–74 years (685 patients), and ≥75 years (221 patients) [10]. The most common indication for surgery in all groups was retrosternal goiter causing compression, although this was least frequent in patients ≥75 years (*P* < .001). Of note, elderly patients were most likely to undergo remedial thyroid surgery (*P* = .003). While hematoma formation was more common in patients aged 61–74 years than those ≥75 years (*P* = .02), the rates of other complications and postoperative death did not differ between groups. Reeve et al. reviewed 575 patients aged >60 years at a single institution [41]. The most frequent operations included thyroid lobectomy (185 cases, 32%) and subtotal thyroidectomy (148 cases, 26%); pathology demonstrated a malignancy rate of 14%. The authors report an “acceptably low” frequency of complications, as the rate of RLN injury and hypoparathyroidism were 1.0% and 0.2%, respectively. However, a total of 68 postoperative events were reported, including hemorrhage requiring reoperation, hematoma, tracheostomy, respiratory problems, myocardial infarction, arrhythmias, and seizures. The authors assert that such general complications “were those expected in an elderly population with multiple pathology”; however, such events highlight the inherent risk assumed by elderly patients undergoing surgery.

 In another single institution series, Rafaelli et al. described the experiences of 320 patients ≥70 years who underwent thyroid surgery [9]. Indications for surgery included bilateral nodular goiter (53.5%), suspicious nodule/confirmed malignancy (28.1%), and toxic goiter (18.4%); 64% of patients presented with ≥1 comorbidity (72.5% of patients of ASA class II). Postoperatively, 23 (7%) patients required ICU admission for management of a concomitant disorder, and average LOS was 3.3 days. Early complications occurred in 39% of patients, including 104 cases of hypocalcemia; hypoparathyroidism and permanent RLN injury were seen in 1.6% and 0.3% of patients, respectively.

 In 279 patients who underwent a total thyroidectomy for MNG at a single institution, Lang and Lo found that patients aged ≥70 years (*n* = 55) were more likely to have retrosternal goiters (76.4% versus 24.4%, *P* < .001), larger goiters (164.1 g versus 100.5 g, *P* < .001), longer operating times (148.9 min versus 136.5, *P* = .023), greater blood loss (102.1 mL versus 66.2 mL, *P* = .030), and fewer parathyroid glands visualized (2.9 versus 3.3, *P* < .001) [35]. While the rate of surgical complications was similar between age groups, the rate of nonsurgical complications was higher in patients ≥70 years (5.5% versus 0.4%, *P* = .021). LOS also was longer among elderly patients (6.4 days versus 3.7 days, *P* < .001). Of note, 31 of 55 elderly patients were ASA class I, 13 were ASA class II, and 11 were ASA class III. Among these individuals, the number of comorbidities was directly correlated to total LOS: 0 comorbidities (4.7 days) versus ≥3 comorbidities (11.0 days).

 An additional review of geriatric thyroidectomy for MNG was performed by Rios et al. The experiences of 591 patients (81 patients aged >65 years) were compared over 30 years [8]. Elderly patients sustained more complications (40% versus 28%, *P* = .011), including transient hypoparathyroidism (*P* = .003) and hematomas (*P* = .034). There were no complications among 8 patients ≥80 years. The authors conclude that surgery for MNG in elderly patients is indicated restrictively.

 Other investigations have focused on the superelderly, or patients ≥80 years. Miccoli et al. reported on 12 patients (mean age 81.4 years) undergoing thyroid procedures for a diagnosis of malignancy or “dramatic” evidence of airway compression [42]. There were no instances of postoperative hemorrhage, RLN injury, or hypoparathyroidism. Mekel et al. described 90 consecutive patients ≥80 years (mean age 83.2 years; range 80–94) who underwent thyroid surgery, and compared them to a randomly selected cohort of 242 individuals aged 18–79 years (mean age 50.1 years; range, 18–79) [43]. No between-group differences were noted with regard to gender, body mass index, previous thyroid operations, principal diagnosis, or final pathology. Of note, octogenarians had higher mean Charlson comorbidity index scores (1.08 versus 0.38, *P* < .001) and ASA scores (*P* < .001). Octogenarians had a higher rate of complications (24% versus 9%, *P* < .001), although there was no difference in the frequency of thyroid-specific complications; there was no mortality in the series. On multivariate analysis, age was not associated with developing a complication. The authors recommend avoiding unnecessary surgery in the elderly, with more strict criteria applied to the biopsy of thyroid nodules.

## 5. Population Studies: Combined Clinical and Economic Outcomes

 While some single institution reports have produced encouraging results regarding the safety of thyroid surgery in the elderly, recent population-level analyses regarding short-term clinical and economic outcomes in geriatric patients have diverged.

 Sosa et al. used the Healthcare Cost and Utilization Project Nationwide Inpatient Sample (HCUP-NIS) to study 22,848 patients undergoing thyroid procedures in the U.S. in 2003-2004; there were 4,092 patients aged 65–79 years and 744 ≥80 years [44]. Older age was associated with a lower likelihood of total thyroidectomy (18–44 y: 44.2%, 45–64 y: 41.1%, 65–79 y: 36.9%, and ≥80 y: 37.1%, *P* < .001 across all ages and procedures), an increased frequency of a substernal component (2.7% versus 3.7% versus 6.3% versus 8.7%, *P* < .001), an increase in the severity of illness (*P* < .001), and an increase in the likelihood of a nonelective hospital admission (*P* < .001). Clear differences in clinical outcomes were noted. In comparison to younger age groups, elderly patients had higher rates of endocrine-specific and overall complications and in-hospital mortality; after adjustment, they also had longer mean LOS and higher costs (all *P* < .001, resp.). These findings were especially pronounced among the superelderly; compared to similar patients aged 65–79 years, patients aged ≥80 years had a 34% increase in complications, a 60% longer inpatient LOS, and a 28% increase in costs. These findings are supported by those of Sosa et al., in their analysis of Maryland Health Services Cost Review Commission data for 5,860 patients undergoing thyroid procedures [45]. After adjustment for patient, surgeon, and hospital characteristics, patients aged ≥70 years had significantly longer LOS, more complications, and greater total hospital charges.

 The SEER-Medicare linked database provides a unique opportunity to follow the incident cancer diagnoses and the subsequent cancer-directed surgeries of Medicare beneficiaries in a SEER registry in a longitudinal fashion, through both administrative records and comprehensive clinical data [46]. Information regarding patient demographics and comorbidities, physician and hospital characteristics, tumor histology, index hospitalization, adjuvant therapy, and complications of treatment are accessible [47]. Tuggle et al. examined the frequency of unplanned hospital readmission within 30 days of discharge among 2,127 elderly patients who underwent thyroidectomy for thyroid cancer in this database [46]. In all, 171 patients (8%) required 185 readmissions, at a mean time of 15.8 days after discharge. Those experiencing a complication during their index hospitalization (22%) were more likely to require an unplanned readmission; the most common reason for rehospitalization was endocrine-specific complications (47%), followed by “other” (25%) and pulmonary complications (17%). The mean LOS for all unplanned readmissions was 3.5 days, with a mean cost of $5,921. Patients who were rehospitalized had a significantly increased risk of death at one year (18% versus 6%, *P* < .001), particularly among those who suffered a pulmonary-related complication (38%). After adjustment, metastatic disease, an increased number of comorbidities, longer index LOS, and having ≥1 postoperative complication at the time of index hospitalization were independently associated with having an unplanned readmission.

 Tuggle et al. also examined the effect of outpatient follow-up on the rate of unplanned readmission. Among the 1,131 patients (53%) seen by a healthcare provider (general practitioner or specialist) after discharge from index hospitalization (mean time to visit: 13.6 days), there was a significantly lower likelihood of readmission than those without prompt physician follow-up (5.1% versus 11.4%, *P* < .001) [46]. As such, the authors emphasize the importance of continuity during the transition from an inpatient to outpatient care setting, especially among elderly patients at high risk for rehospitalization.

## 6. Discussion

 Surgical management of elderly patients presents a unique set of challenges, including overcoming compromised access to appropriate care and high-volume surgeons, and increased patient complexity and severity of illness [44]. To date, the majority of studies regarding outcomes for elderly patients undergoing thyroid surgery have been largely limited to the single institution experiences of high-volume, tertiary care centers. It is not surprising that many of these small series have demonstrated superlative outcomes; previous population-level analyses have delineated a positive association between increased surgeon and hospital case volume and patient outcomes, including those performed on children [48], pregnant women [49, 50], and the elderly [51]. For thyroid surgery in an elderly population, the use of a representative national database supports this notion [44, 45]. Therefore, small published case series may underestimate the true risks of surgery for the average elderly patient with thyroid pathology, potentially limiting external validity.

Using the HCUP-NIS database, Sosa et al. found that, among all age groups (18–44 y, 45–64 y, 65–79 y, and ≥80 y) surgeons performing >100 thyroidectomies/year had shorter LOS and lower complication rates than their lower-volume colleagues [44]. When patients were stratified by age and comorbidities, elderly and superelderly patients who underwent thyroidectomy by high-volume surgeons (≥30 cases/year) had shorter LOS and lower-complication rates than patients who had surgery by low-volume surgeons; high-volume surgeons also had lower total costs in all groups except healthy superelderly patients. These results were particularly pronounced among superelderly patients with multiple comorbidities, where high-volume surgeons had lower rates of complications than their low-volume colleagues (11% versus 25%), shorter LOS (2.2 versus 7.7 days), and lower total costs ($5,140 versus $12,541).

Despite these findings, low-volume surgeons continue to perform the overwhelming majority of thyroid surgery, particularly among elderly patients. Using data from the 1988–2000 HCUP-NIS, Saunders et al. found that 82% of all thyroidectomies were performed by surgeons with a practice composed of <25% endocrine procedures; surgeons with a practice consisting of >75% endocrine procedures performed just 3% of all thyroidectomies [52]. Sosa et al. found that not only did lowest volume surgeons (1–9 cases/year) account for the largest proportion of thyroidectomies among all patient age groups examined, but that this trend increased with patient age (18–44 y: 47%, 45–64 y: 46%, 65–79 y: 52%, and ≥80 y: 58%, *P* < .001); for surgeons performing >30 thyroidectomies/year, the reverse trend was noted (18–44 y: 29%, 45–64 y: 29%, 65–79 y: 23%, and ≥80 y: 16%, *P* < .001) [44]. This is a finding of concern, and may partially explain why the outcomes of elderly patients undergoing thyroid surgery on a national scale appear to be inferior to those from select high-volume institutional cohorts.

A majority of published single-institution case series have found no effect of age on the risks of thyroid surgery. However, given their small sample sizes and thus the risk of associated type II error, it is possible that these studies have been underpowered to adequately test the hypothesis of interest. Underpowered studies, which frequently fail to calculate or report power analyses, are prevalent in the surgical literature [53–55]. In a review of randomized control trials in the surgical literature, Maggard et al. found that only 38% reported sample size calculations; among all studies, 81% did not have a large enough sample size to detect a 20% outcome difference, with 55% of the these studies requiring a 10-fold increase in sample size [55].

Observational studies assessing comparative effectiveness or safety of different treatment strategies can suffer from confounding by indication [56]. Analytic tools such as propensity scores are being employed more and more in surgical health services research to account for residual selection bias, especially when the outcomes of interest are rare, as morbidity and mortality can be in endocrine surgery [57]. As evidence-based practice continues to drive clinical and operative decision-making, it is critical to understand the strength of conclusions to be drawn from studies regarding surgical outcomes.

Thyroid surgery presents distinct risks for elderly patients; on a national scale, the clinical and economic outcomes for many of these individuals appear to be compromised compared to younger patients. Thyroid surgery in this population should be applied selectively, and undertaken after meticulous preoperative medical optimization. Given the correlation between surgeon caseload and postoperative morbidity, strong consideration should be given for referral of elderly patients to high-volume surgeons, especially among individuals with complex or malignant thyroid pathology. Attention also should be paid to ensure the surgical care of elderly patients follows current practice guidelines, and aggressive postoperative follow-up should be standardized to minimize the risk of costly, unplanned hospital readmissions. Future areas of analysis could include a further clarification of indications for thyroid surgery in this population, as well as outcome measures such as long-term survival and quality of life.
